# A primary health care Re-Engineering approach to enhance PrEP initiation and adherence among high-risk, sexually active adolescents and young adults in KwaZulu-Natal, South Africa

**DOI:** 10.1186/s12889-025-25698-2

**Published:** 2025-12-22

**Authors:** Thubelihle Mathole, Jeannine Uwimana Nicol, Mbuzeleni Hlongwa, Eunice Turawa, Wisdom Basera, Noluntu Funani, Edward Nicol

**Affiliations:** 1 Burden of Disease Research Unit, South African Medical Research Council, Cape Town, South Africa; 2 School of Public Health, University of the Western Cape, Cape Town, South Africa; 3 Epidemiology and Biostatistics Division, Stellenbosch University, Cape Town, South Africa; 4 School of Public Health, University of Rwanda, Kigali, Rwanda; 5 Public Health, Societies and Belonging, Human Sciences Research Council, Pretoria, South Africa; 6 School of Nursing and Public Health, University of KwaZulu-Natal, Durban, South Africa; 7 Division of Health Systems and Public Health, Stellenbosch University, Cape Town, South Africa; 8 School of Public Health and Family Medicine, University of Cape Town, Cape Town, South Africa

**Keywords:** Primary health care, Pre-exposure prophylaxis, PrEP uptake, Sexual and reproductive health, Sexually active, Service delivery points, AGYW, ABYM

## Abstract

**Objective:**

This study explores the decentralisation of PrEP services through primary health care (PHC) re-engineering structures such as schools, pharmacies, youth zones, community halls, and mobile clinics to improve PrEP uptake and adherence among adolescents and young adults (AYAs) in KwaZulu-Natal, South Africa.

**Methods:**

In-depth interviews were conducted from August 2021 to July 2023 with 48 purposively selected participants from a cohort of 2,772 newly diagnosed HIV-negative, sexually active, high-risk individuals. These included 36 adolescent girls and young women aged 15–24 years and adolescent boys and young men aged 15–35 years who initiated PrEP within one month at various service delivery points, including clinics, schools, and community-based services. An additional 12 participants who had not initiated PrEP were also included. Data were analysed thematically using a comprehensive codebook developed to guide the coding process. All transcripts and audio recordings were validated for completeness and accuracy before coding.

**Findings:**

The study identifies critical factors that support successful PrEP implementation and expansion among high-risk, sexually active AYAs. The PHC re-engineering programme emerged as a crucial strategy for increasing both PrEP uptake and adherence. Participants expressed a strong preference for decentralised service models, including community-based facilities, outreach teams, and home delivery systems. These models were most appealing to AYAs compared to traditional healthcare facilities. They effectively addressed common barriers such as long waiting times, unfriendly healthcare professionals, overcrowding, stigma, and transportation challenges. Some participants noted that home delivery of PrEP saved both time and money, contributing to better adherence.

**Conclusion:**

Decentralised, community-based approaches play a vital role in improving PrEP uptake, adherence, and continuation among AYAs in South Africa. The findings underscore the importance of addressing key barriers such as distance, stigma, and accessibility. By decentralizing PrEP services and embedding them within familiar, youth-friendly spaces at community level, this study demonstrates how HIV prevention can be made more accessible and acceptable to AYAs.

**Supplementary Information:**

The online version contains supplementary material available at 10.1186/s12889-025-25698-2.

## Background

The Human Immunodeficiency Virus (HIV) remains one of the most pressing global health challenges, with South Africa bearing the highest burden of HIV infection in the world. As of 2023, an estimated 7.7 million people, approximately 12.5% of the population, are living with HIV [1]. While recent years have seen a decline in new HIV infections [2], adolescents and young adults (AYAs) remain disproportionately affected by the epidemic. Individuals aged 15–24 face a significant risk of HIV infection compared to the older age groups, underscoring the need for targeted interventions to address their unique vulnerabilities [3].

Adolescence is a critical development stage marked by increased susceptibility to HIV infection among both adolescent girls and young women (AGYW) and adolescent boys and young men (ABYM). During this stage, individuals experience stress, challenges and transitional changes that can affect their future aspirations and, consequently, the socio-economic landscape of South Africa [3]. Factors such as limited access to healthcare services, high rates of unintended/unplanned pregnancies, and pervasive gender-based violence further exacerbate these vulnerabilities [4]. Additionally, cultural norms and societal expectations often restrict young people’s access to sexual and reproductive healthcare services, further limiting their ability to make informed decisions about HIV prevention and care [5].

PrEP is an HIV prevention strategy that has been shown to reduce the risk of HIV acquisition by 99% if taken as prescribed [6]. The South African government has made significant strides in prioritising the rollout of Pre-Exposure Prophylaxis (PrEP) as a key component of its HIV prevention strategies. Initially introduced in 2016 across five provinces, PrEP was first made available to selected key populations, including sex workers, men who have sex with men, and AGYW [7]. Since then, it has become an essential tool in the fight against HIV transmission, particularly among high-risk populations such as AGYW and ABYM [8].

One of the key models supporting the implementation of PrEP is the Determined, Resilient, Empowered, AIDS-free, Mentored, and Safe (DREAMS) initiative, launched in 2014 by the U.S. President’s Emergency Plan for AIDS Relief (PEPFAR) [9]. DREAMS targets high-risk AGYW, who remain disproportionately affected by the HIV epidemic, by addressing structural drivers of HIV vulnerability, including gender inequality, poverty, and limited access to healthcare services.

In addition to DREAMS, South Africa has adopted several innovative approaches across various service delivery points (SDPs) to enhance PrEP uptake and continuation among adolescents. These include the establishment of community-based drop-in centres and the integration of PrEP services into existing platforms such as family planning, maternal and child health, sexual and reproductive health, and antenatal care services [10]. Furthermore, the introduction of adolescent-friendly clinics and community-based, youth-friendly delivery modalities has played a pivotal role in making PrEP services more accessible, acceptable, and responsive to the needs of young people [11].

Complementing these targeted interventions, South Africa also developed the Primary Health Care (PHC) re-engineering strategy as part of broader efforts to strengthen PHC service delivery under the framework of the National Health Insurance (NHI). This strategy is informed by both local health system challenges and global lessons from countries that have successfully implemented similar models. The PHC re-engineering programme is mostly composed of Ward-based Primary Health Care Outreach Teams (WBPHCOTs), which mainly comprises of school health teams and district clinical specialist teams [12]. These teams play a critical role in extending preventative and promotive health services into communities and serve as an important platform for integrating interventions such as PrEP at the community level.

The PHC re-engineering programme aims to shift the health system from a predominantly curative, vertical, and individual-oriented model toward a proactive, integrated, and population-based approach to healthcare delivery [13]. It is designed to address health promotion, disease prevention, and rehabilitation needs at the community level [14]. By decentralising healthcare services and integrating them within communities, through outreach teams, schools, colleges, youth zones, pharmacies, community halls, and mobile clinics, the PHC re-engineering programme seeks to reduce barriers to healthcare access, particularly for underserved and high-risk populations. Implemented as a key component of broader health sector reforms, the PHC re-engineering strategy reflects South Africa’s commitment to achieving universal health coverage and provides a critical platform for delivering integrated health interventions at the community level [12, 14].

The challenge with access to healthcare is further compounded by systemic issues such as medicine stockouts and long waiting times [15]. For South African youth, these obstacles are further intensified by stigma and discrimination from some healthcare providers, which continue to undermine access to and uptake of PrEP. In addition to healthcare system limitations, young people face structural and socio-economic barriers that hinder their ability to engage with HIV prevention services. These include high rates of poverty and unemployment, deeply entrenched cultural norms and gender dynamics [16], and clinic operating hours that are often misaligned with the daily lives of adolescents and young adults [8].

Although, the PHC re-engineering programme was introduced in 2016 [17], there remains a limited body of literature on the use and effectiveness of decentralised services in improving healthcare access among AYAs. While certain components like District Specialist Teams and the utilisation of Community Health Workers are relatively well documented [18, 19], other key structures of PHC re-engineering, including Ward-Based Outreach Teams and community-based services, remain under-utilised and under-researched. This study explores the decentralisation of PrEP services through PHC re-engineering structures - including schools, pharmacies, youth zones, community halls, and mobile clinics to enhance PrEP uptake and adherence among AGYW and ABYM in KwaZulu-Natal, South Africa.

## Methods

### Study design and setting

A qualitative study design was employed to explore participants’ perceptions and experiences regarding the decentralisation of PrEP services using PHC re-engineering structures at community level to improve the initiation, continuation, and adherence to oral PrEP. This study was conducted from August 2021 to July 2023 as part of a broader research initiative on identifying effective and feasible PrEP models of care for improving PrEP uptake, continuation, and adherence among AGYW and ABYM in South Africa [20]. It was conducted across 22 service delivery points (SDPs) in uMgungundlovu district, KwaZulu-Natal province, South Africa, a district that has a high HIV prevalence, accounting for 24% and 37% of people aged 15–49 years in males and females, respectively [21]. The district is one of the NHI pilot sites, with 46 fixed clinics (primary health care facilities or community health centres), and 17 mobile clinics. The majority of the population in uMgungundlovu district is predominantly poor and resides in rural areas, relying on public healthcare services [22].

### Study population

The study population included sexually active adolescent girls and young women (AGYW) aged 15–24 years, as well as adolescent boys and young men (ABYM) aged 15–35 years, who tested HIV negative at 12 selected SDP for this phase of the study. These SDPs comprised four routine facility-based sites, three school-based sites, and four community-based youth zones, across all seven sub-districts of the uMgungundlovu district.

### Sample size with a subset of participants

A total of 48 participants were purposively drawn from a cohort of 2,772 newly identified HIV-negative, sexually active, high-risk individuals. Participants were classified as high risk if they met any of the following criteria: (i) reported having multiple sexual partners, (ii) received a positive diagnosis for a sexually transmitted infection (STI) within the preceding six months, (iii) had a partner known to be HIV-positive, (iv) reported inconsistent condom use, or (v) did not know their partner’s HIV status.

The sample included 36 AGYW and ABYM who had initiated PrEP within one month at various service delivery points, including clinics, schools, and community-based Youth Zones [23], as well as 12 participants who had not initiated PrEP. Selection was based on 1-month outcome data and was stratified by age, gender, and type of service delivery model.

For analysis, participants were further grouped to capture different stages of PrEP care continuum: 12 participants who initiated PrEP at enrolment, regardless of whether they continued or discontinued afterward. 12 participants who were eligible but chose not to start PrEP, 12 participant who had maintained PrEP use consistently for 4 months after initiation, and 12 participants who had been on PrEP but discontinued by 7 months. This approach allowed for a comprehensive understanding of young people’s experiences and perspectives across different stages of the PrEP continuum.

### Data collection procedure

A qualitative interview guide (Supplementary file 1), developed in collaboration with experienced senior investigators, was piloted with a small number of participants from the target population prior to data collection. The pilot ensured that questions were clear, culturally appropriate, and relevant, and informed minor revisions to improve comprehension and flow. In-depth interviews (IDIs) were then conducted with a purposive subset of participants in English or isiZulu, the predominant language in the uMgungundlovu district. We conducted 12 IDIs per each of the four PrEP groups—those who initiated PrEP within 1 month, those who did not, those who continued PrEP at 4/7 months, and those who discontinued resulting in 48 interviews. The IDIs were conducted at the participants’ homes or clinics, based on their preference.

Topics explored included participants’ knowledge and awareness of PrEP, perceptions of HIV risk, decisions around PrEP initiation, continuation, or discontinuation, adherence challenges, access to services, interactions with health providers, community and social support, and suggestions for improving PrEP delivery and communication strategies. This approach allowed for an in-depth understanding of participants’ experiences across different stages of the PrEP care continuum, while the full interview guide is available in the supplementary material.

This method also examined participants’ perceptions and experiences with different PrEP delivery models, focusing on their interactions with and the perceived usefulness of each model in enhancing engagement with PrEP. Norms and attitudes regarding PrEP delivery and implementation were also explored. Participants provided informed consent for IDIs, which lasted 60–120 min depending on the depth of interaction. The interviews were audio-recorded, transcribed verbatim in the local language, before being translated into English. Each transcript and recording were validated for completeness and accuracy by a bilingual senior research team member before coding was performed. Audio recordings were, identified only by enrolment number, was uploaded daily to REDCap, via 3G or Wi-Fi from facility-based tablets to the password-protected SAMRC-hosted REDCap server.

### Data analysis

Data analysis was conducted manually by two of the co-authors trained in qualitative research methods. Following the principles of thematic analysis outlined by Braun and Clarke (2006) [24], both two authors independently read and open-coded interview transcripts to identify how decentralisation of PrEP services through PHC re-engineering structures influenced PrEP uptake and adherence among AYAs. All qualitative transcripts were coded to identify emerging themes aligned with study objectives. A comprehensive codebook was developed based on the study objectives, and two coders independently analysed the data using both deductive and inductive approaches [25, 26]. Regular team meetings were held to discuss codes refinement and ensure analytical consistency. Discrepancies between coders were resolved through consultation with all members of the research team until consensus was achieved.

Theme development was guided by the study’s research questions, objectives and interview guides, ensuring alignment between the analytical framework and study aims. Emerging themes were iteratively reviewed, refined, and compared both within and across cases to deepen understanding of the data [27]. Preliminary themes were subsequently presented to the research team for discussion and validation, which informed additional analysis and generation of the final themes. Team members were further consulted to reach a consensus on data interpretation. Unclear issues were clarified by study participants and data collectors to ensure accuracy.

## Findings

### Demographic characteristics

Table 1 outlines the demographic characteristics of 48 participants interviewed between August 2021 and July 2023 from 11 service delivery points. All were Black Africans, with 69% recruited from clinics and 13% from gateways. Most were female (67%) and in relationships (77%). Additionally, 92% were in high school, 69% were unemployed, 67% experienced low food security, and 65% received child support grants.

**Table 1:** Socio-demographic characteristics of AGYW and ABYM disaggregated by PrEP initiation and continuation at uMgungundlovu district between August 2021 to July 2023

	Total *n*=48, (%)	Initiated PrEP at 1 month *n*=12, (%)	Continued PrEP at 4/7 months *n*=12, (%)	Discontinued PrEP at 4/7 months *n*=12, (%)	Did not initiate PrEP at 1 month *n*=12, (%)
Nationality					
South African citizen	48 (100)	12 (100)	12 (100)	12 (100)	12 (100)
Ethnicity					
Black African	48 (100)	12 (100)	12 (100)	12 (100)	12 (100)
Facility type					
Mobile clinic	2 (4)	0	0	1 (8)	1 (8)
Clinics	33 (69)	10 (84)	7 (59)	9 (75)	7 (58)
CHC	3 (6)	1 (8)	1 (8)	1 (8)	0
Gateway	6 (13)	1 (8)	3 (25)	1 (8)	1 (8)
Other	4 (8)	0	1 (8)	0	3 (25)
Sex					
Male	10 (33)	4 (33)	3 (25)	1 (8)	2 (17)
Female	38 (67)	8 (67)	9 (75)	11 (92)	10 (83)
Education level					
Primary education	1 (2)	0	0	0	1 (8)
High school education	44 (92)	12 (100)	10 (83)	11 (92)	11 (92)
Post matriculation	3 (6)	0	2 (17)	1 (8)	0
Age categories					
15-19 years	10 (21)	2 (17)	2 (17)	3 (25)	3 (25)
20-24 years	31 (65)	9 (75)	8 (67)	8 (67)	9 (75)
25-29 years	4 (8)	1 (8)	2 (17)	1 (8)	0
Marital status					
Cohabiting	1 (2)	0	1 (8)	0	0
Dating	36 (75)	12 (100)	9 (75)	8 (67)	7 (58)
Single	10 (21)	0	1 (8)	4 (33)	5 (42)
Prefer not to answer	1 (2)	0	1 (8)	0	0
Service delivery point (SDP)					
Clinics	11 (23)				
Gomane Clinic	2 (4)	0	0	1 (8)	1 (8)
Maguzu Clinic	3 (6)	2 (17)	0	0	1 (8)
Mphophomeni Clinic	1 (2)	0	1 (8)	0	0
Moor river Clinic	5 (10)	3 (25)	0	0	2 (17)
Youth Zones	26 (54)				
Richmond Youth Zone	7 (15)	0	3 (25)	1 (8)	3 (25)
Taylors Youth Zone	1 (2)	1 (8)	0	0	0
Caluza Youth Zone	6 (13)	2 (17)	1 (8)	2 (17)	1 (8)
Imbalenhle Youth Zone	12 (25)	3 (25)	3 (25)	4 (33)	2 (17)
TVET Colleges	11 (21)				
Applesboch TVET	9 (19)	1 (8)	4 (33)	2 (17)	2 (17)
Richmong TVET	1 (2)	0	0	1 (8)	0
High Schools	1 (2)				
Phayapini High School	1 (2)	0	0	1 (8)	0
Mode of transport to SDP					
On foot	23 (48)	5 (42)	3 (25)	6 (50)	9 (75)
Public transport	17 (35)	4 (33)	8 (67)	4 (33)	1 (8)
Private transport	8 (17)	3 (25)	1 (8)	2 (17)	2 (17)
Time to get to SDP					
Less than 30 minutes	40 (83)	12 (100)	7 (58)	11 (92)	10 (83)
30-60 minutes	7 (16)	0	4 (33)	1 (8)	2 (17)
More than an hour	1 (2)	0	1 (8)	0	0
Worked in the last 12 months					
Never worked​	33 (69)	7 (58)	9 (75)	7 (58)	10 (83)
Once in a while​	12 (25)	4 (33)	2 (17)	4 (33)	2 (17)
Most months​	3 (6)	1 (8)	1 (8)	1 (8)	0
Food insecurity*					
Low	35 (67)	8 (67)	8 (67)	8 (67)	11 (92)
Medium	13 (33)	4 (33)	4 (33)	4 (33)	1 (8)
Access to US$14 in emergencies					
Somewhat/very difficult	31 (35)	6 (50)	8 (67)	9 (75)	8 (67)
Fairly/very easy	17 (65)	6 (50)	4 (33)	3 (25)	4 (33)
Received child support grant					
Yes	31 (65)	8 (67)	8 (67)	7 (58)	8 (67)
No	17 (35)	4 (33)	4 (33)	5 (42)	4 (33)

Proportions (%) for the columns reported as n/N

Community health centre (CHC) is a facility that typically offers primary health care (PHC) services, including 24-hour maternity care, accident and emergency services and observation beds for patients up to 48 hours. It usually has a procedure room but not an operating theatre

Clinic is a facility providing a variety of PHC services and is normally open for eight or more hours a day, depending on the community’s needs

AGatewayis a PHC clinic located within a CHC or a hospital’s Out-patient department (OPD). It caters to patients with minor ailments who are treated by trained PHC workers at no charge, before being referred to a CHC or hospital. Each CHC/ hospital has a gateway clinic attached to it to serve the local population

*US$* United States of American Dollar, *PrEP * Pre-exposure prophylaxis, *PHC * Primary health care, *CHC*–Community health centre, *TVET* Technical and Vocational Education and Training

^*^Food security included questions about whether in the last four weeks the participant or other household members lacked food, went to sleep hungry or borrowed

### PHC re-engineering structures

**Fig. 1:**
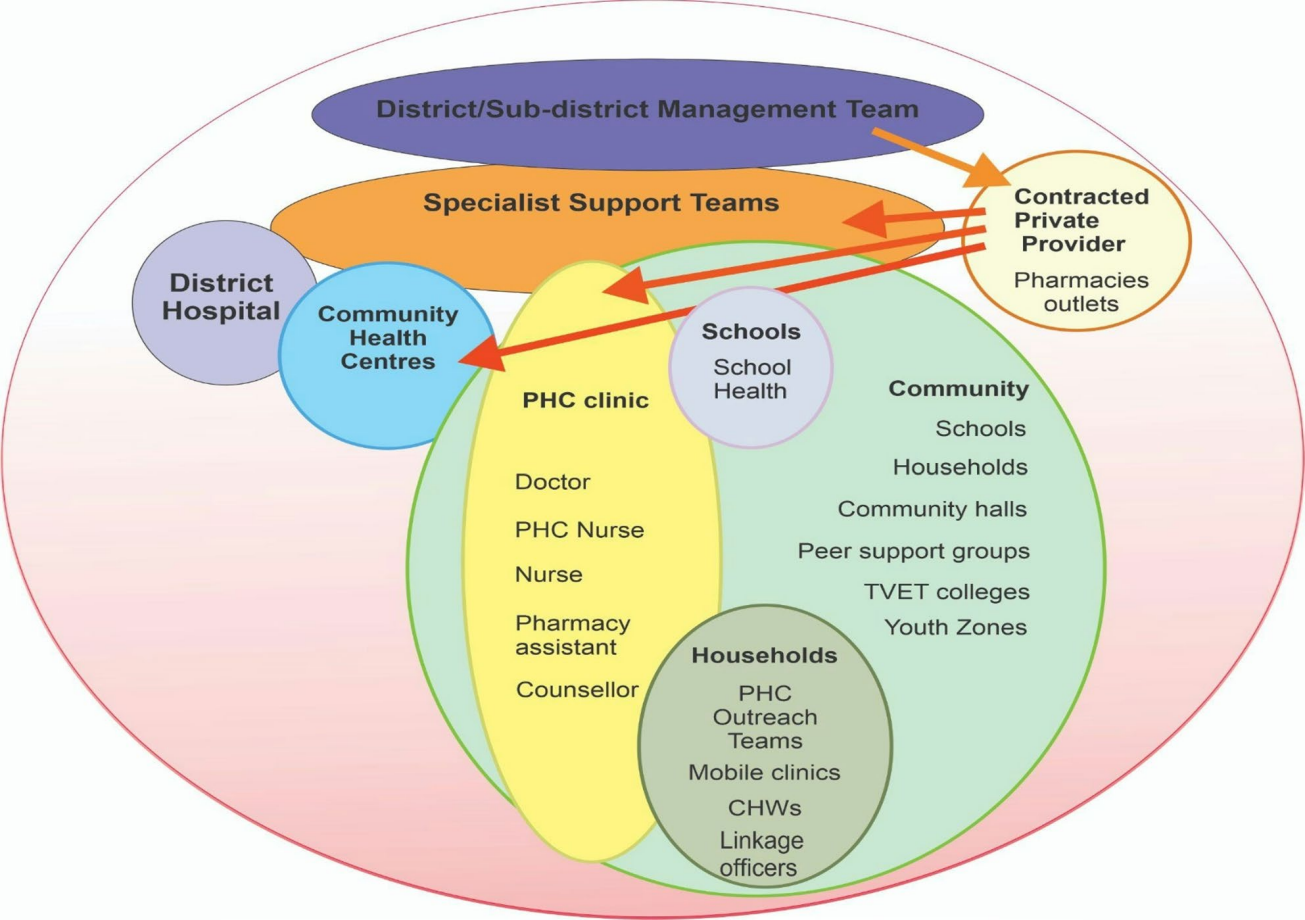
Adapted from the proposed PHC Re-Engineering approach (Yogan Pillay and Peter Barron 2010) ^[12]^

Our data demonstrate how the PHC re-engineering model can be used to increase the uptake and adherence of PrEP in low-resource settings. The Fig. 1 shows a diagrammatic presentation of the PHC re-engineering model at a district level [12]. The model involves decentralizing and simplifying services by moving them out of a traditional health facility setting and decentralising them through schools, health services programme, and outreach teams to local communities to increase access of PrEP services and collection of PrEP pills from community-based service points like schools, colleges, pharmacies, community halls and others.

Thematic analysis identified two main themes, eight sub-themes, and nine categories, which indicates varied preference among participants regarding PrEP service delivery points (Table 2). Some participants favoured healthcare facilities, citing easy access (particularly for those living close to health facilities) and the presence of friendly staff. Others, however, preferred decentralized and simplified service delivery models, noting both the benefits and challenges of accessing different service delivery points. Overall, most participants expressed a preference for PrEP provision in schools, while pharmacies, health facilities, mobile clinics, community halls, NGO facilities and other outreach teams were also identified as acceptable collection points or service points for PrEP. Across the discussion, participants emphasized the importance of decentralized, easily accessible service points.

**Table 2:** Overview of themes and subthemes relating to adolescents and young adults’ perceptions about varied preference regarding PrEP service delivery points

Themes	Sub-Themes
Provision of PrEP at Primary Health Facilities	Preference of Health Facilities
	Negative Experiences in Health Facilities • Long queues and long waiting times • Staff Attitudes and Cultural beliefs/Fear to be judged and ridiculed • Nurse being part of the culture • Lack of privacy and Confidentiality. • HIV related Stigma • Stigma associated to use of Park Homes
Community Based Service Providers	Schools and Colleges • Accessible (distance and transport cost) • Peer support • Health promotion - Share information with friends
	Pharmacies
	Community Halls
	Community Health Workers and Support Groups
	Youth Zones
	Home Delivery

### Provision of PrEP at health facilities

#### Preference of health facilities

We assessed perceptions of participants, what they like and dislike about provision of oral PrEP in clinics. Participants had mixed views on the use of clinics as a service delivery point for PrEP. Participants that stayed close to health facilities preferred using them as collection points because of geographic proximity, privacy and friendliness of staff as indicated by one of the participants who said that it was:

*“…easier to get to the clinic”* *(GOCHIM070_P_D4-7M_F_20).*

Similar sentiments were shared by other participants who explained how the short distance to the clinic made it easier for them to access PrEP services:

*“It’s fine with me since I don’t have to use any transport to get here”(GOCHIM251_P_C4-7M_M_25).*

*“I walk to the clinic. I do not have to use transport to the clinic*,* so it is easy to get to it”* *(CAYCMS020_P_I_F_23).*

When asked about going to another clinic for PrEP, a 24-year-old PrEP user stated:

*“This clinic is close. I won’t lie and say I’d not continue. I’d be lazy because other clinics are far”* *(RITSRI018_P_C4-7M_F_22).*

When asked generally about the advantage of getting PrEP services at the local clinic, one of the participants stated that

*“…they have made my life easy…I am able to come and collect PrEP on my appointment date …”* *(IMYCMS037_P_C4-7M_F_23).*

Besides geographic proximity of the clinic to participants, use of appointment system, respect, privacy, informative and friendly staff were also mentioned as some of the factors that made some of them prefer the clinic. One of them said:

*“…now I don’t mind taking PrEP at the clinic*,* the clinic is close to my home*,* and I know all the nurses*,* they are friendly to me and respectful. They are also teaching us about PrEP…”* *(IMYCMS069_P_D4-7M_F_25).*

Another reason for preferring the health facility was that at:

*“… the clinic you can get treatment for all other diseases as compared to a pharmacy.”* *(MOCHMP119_P_I_F_22).*

Stigma and fear of disclosure about the use of PrEP to family and neighbours resulted in some of the adolescents and young adults preferring to use clinics for PrEP services instead of home deliveries as they fear being seen or known that they are taking PrEP pills. One of the participants stated:

“*Mm*,* but I think it would be better to get PrEP at the clinics or pharmacies because … it won’t be too obvious that you may be going to get PrEP pills… Someone else might think that maybe you have a headache*,* or any other problem and you are just going to the clinic for that. I do not want people to know that I take PrEP… I did not tell my family; I did not tell anyone that I am taking PrEP.”* *(IMYCMS062_P_I_M_18).*

Similar sentiments were shared by other participants:

*“People go … to the clinic*,* rather than having a mobile clinic delivering PrEP pills at the gate. I do not like mobile clinics because neighbours will see me… It attracts attention [laughing]”* *(APTSUM066_P_C4_7M_F_23).*

*“In my opinion I think it is better at the Clinic*,* because maybe you will find that we are just sitting at home and the CHW or the mobile van delivers pills and that will make people to talk and say that ‘hhaybo [an expression of surprise or disbelief] they are delivering HIV drugs*,* and start asking you questions [laughing]. It is better to collect it [PrEP pills] at the clinic*,* … and come back home; no one will see you…people talk too much.”* *(MOCHMP017_P_D4-7M_F_22).*

#### Negative experiences in health facilities

Although PHC clinics were identified as one of the preferred PrEP delivery points, some of the participants, however had different experiences using the clinics especially those who travelled long distances to the facilities highlighting challenges of lack of transport money as expressed by some of the participants:

*“…the clinic is very far from us*,* and it is difficult*,* and you need money for transport to go and collect your PrEP pills”* *(CAYCMS003_P_DNI_M_24).*

Some participants were not happy with facility operating times and the attitude of some staff members as expressed by one participant:

“*At our clinic staff members are rough and rude*,* right from the security guard*,* the lady that gives you your card at times they chase you and tell you are late*,* but I was at school*,* what should I do.*,* the nurses will at times ask you why are you having sex when you are so young… it’s not good…”* *(APTSUM066_P_C4_7M_F_23).*

Participants highlighted fear of HIV stigma, misconceptions about PrEP, fear of being judged and a lack of privacy at health care facilities, as challenges to accessing PrEP services at the healthcare facilities.

“*The good thing is that a clinic is in a place that is close to us*,* that we usually visit…as a community*,* but the bad thing is that people judge each other*,* some will end up self-conscious…there is no privacy*,* when people see you taking PrEP pills they think you have got HIV*,* you are sick and they talk bad things about you*” *(MOCHMP017_P_D4-7M_F_22).*

##### Long queues and long waiting times

Poor living conditions in our study area, distance to clinic, long queues and long waiting times were listed by our participants as factors that contribute to their reluctance to use health facilities for PrEP services as stated below:

“…*some of us do not like Clinics*,* you travel long distance to go to the clinic*,* you get there you join long queues just to collect a pill*,* I do not like that at times you miss your bus back home because you are still waiting in the clinic*,* just imagine…*” *(IMYCMS037_P_C4-7M_F_23).*

*“…I no longer go to the clinic…the nurse will shout at us if we do not go and collect our PrEP pills at the clinic because they don’t know the condition of the people i.e. where we sleep*,* what we eat*,* the distances we travel… we do not have money to pay for transport….”* *(MOCHMP017_P_D4-7M_F_22).*

*“… it is very far*,* and it gets full*,* with long queues…so the mobile clinic helps because they also have it in the community*,* …ehh there is no line …”* *(CAYCMS020_P_I_F_23).*

#####  Staff attitudes and cultural beliefs/fear to be judged and ridiculed

The study identified significant discomfort with the healthcare system’s handling of sexual health issues. Cultural and religious beliefs within the community influenced how healthcare workers approached adolescent sexuality. Some workers reportedly discriminated against young individuals seeking PrEP services, with instances of scolding and stigmatizing behaviours. These negative attitudes not only discouraged adolescents from accessing PrEP but also highlighted a potential barrier to care, as healthcare providers with such beliefs may withhold or fail to adequately offer PrEP to those who need it as expressed by some of the participants in the quotes below:

*“Definitely not the Clinics*,* I will not go to the clinic… most of us complain that nurses are rude*,* they are judgmental you know… they will tell you that you can’t have sex at such a young age*,* and they will not give you PrEP pills…they will tell you to abstain”* *(IMYCMS021_2_P_I_F_21).*

*“…I think it’s a good idea because some students are afraid to go to hospitals*,* they are not comfortable with nurses who ask too many questions about their sexual life… they shout at you if you are young*,* and you are sexually active.”* *(APTSUM044_P_C4_7M_M_24).*

*“…I no longer go to the clinic…the nurse will shout at us if we do not go and collect our PrEP pills at the clinic…”* *(MOCHMP017_P_D4-7M_F_22).*

*“They [nurses] will just turn you back if you come after 1.00 pm and advise you to come on another day. You have to wake up early to join the queue before 7.00 am*,* …but we are in school. So*,* what do you do?”* *(APTSUM124_P_I_M25).*

Participants described how the negative attitudes of some healthcare staff resulted in confusion and a lack of trust in clinic-based services. Such experiences led to fear when interacting with nurses, prompting participants to suggest that PrEP services be offered in schools because:

*“… it’s not easy to go to the clinic*,* because it is very far. It’s better for us if you just come here to our school*,* we do not have to travel… There are many people who are afraid (stutter) to go to the clinic and the hospital… they fear nurses who shout at them.”* *(APTSUM124_P_I_M25).*

The negative attitudes of clinics staff contributed to fear and reluctance among adolescents to access PrEP service at healthcare facilities. Participants described some nurses as being ‘rough’ or unapproachable. Additionally, adolescents expressed concerns that being seen accessing PrEP services at the clinic could lead to judgement and stigma from community members who might assume they are living with HIV, as illustrated in the quote below:

*“…it’s better if the people…the PrEP people come to our school because there are people who are afraid to go to the hospital. They fear the nurses who are rough… and they fear to be seen by the people we know in the clinic; people talk too much*,* they think if you take pills*,* you are sick with HIV…”* *(MACHMK088_P_D4-7M_F_22).*

##### Nurse being part of the culture

Cultural norms and values influence how health professionals approach and manage young women and men in PrEP. This study was conducted in one of the provinces where African cultural norms and practices are religiously practiced and early debut in sex practice by girls is discouraged:

*“The disadvantage of a clinic is that if you are late in collecting PrEP*,* the nurses will be disrespectful and shout at you for not taking your life seriously.”* *(MOCHMP119_P_I_F_22).*

*“I won’t comment that much but I think it’s good to deliver PrEP at school because there are lots of kids of the same age that will support you and share experiences but at the clinics there the older nurses end up mistreating you and judging you for what you are doing because they think you are too young to have sex.”* *(TAYCMS033_P_I_F_22).*

Our data also highlight the challenges of the age gap between the health care workers and the young people – and its influence on how they manage young people accessing PrEP services. Some participants for example preferred young nurses because they relate well in discussing sexual issues, and were not comfortable to discuss these with older nurses as shown in the quotes below:

*“…uh I prefer the nurse to be at our age group that I will be able to speak to her. They mustn’t be old. (laughs) …I will be more comfortable because a person in our age group may be able to answer me in a better way… A grown person will tell me what my mother tells me*,* they will tell me to stop having sex because I am too young*,* that is what all these old mamas in this area believe…”* *(MOCHMP017_P_D4-7M_F_22).*

*“… at the clinics there the older nurses end up mistreating you and judging you for what you are doing because they think you are too young to have sex.”* *(TAYCMS033_P_I_F_22).*

##### Lack of privacy and confidentiality

Due to lack of adequate infrastructure in some facilities, consultation rooms are shared among healthcare workers, making it difficult to maintain privacy and confidentiality during consultations as highlighted in the quote below:

*“The problem with the clinic is that they use one room for two nurses*,* they just divide it with a curtain*,* so when they ask you personal questions*,* the other people in the room will be listening… you feel embarrassed and humiliated. I can’t even ask other personal questions because you will never know who is at the other side of the curtain…”* *(IMYCMS140_P_D4-7M_M24).*

##### HIV related stigma

The use of the same patient identification cards (card colour) for both HIV and PrEP clients created a level of fear of being stigmatised and identified as a HIV patient as expressed by one of the participants:

*“Because people are scared to go at clinics with their blue cards because they are the same with the cards of those that are HIV positive*,* people will think you are HIV positive*,* it’s better if we do not carry the card*,* it better if we will show it inside where you are not seen by people. So*,* they should allow us to only use our ID number*,* and they should take the file to the sister then we queue without a card.”* *(MOCHMP119_P_I_F_22).*

Besides the use of similar identification cards for both HIV and PrEP clients, some of the participants did not want to be seen at the park homes designated for HIV care.

##### Stigma associated to use of park homes

Due to limited infrastructure in most health facilities in rural areas, Park homes (temporal structures) that were brought in to create more space and accommodate high numbers of HIV patients in public health facilities was also described as a barrier to access of PrEP services. Park homes were used for HIV services only, thereby isolating HIV patients from the rest of the patients and – exacerbating stigma in this group. Some participants did not want to be seen in those queues, and they complained that:

*“…they separate us at the clinic*,* and we collect our PrEP pills from the containers (Park Homes)*,* you know those containers used by HIV people at the back*,* I do not like going there because when people see us there*,* they think we are all HIV positive*,* they start talking about us and judge us. So*,* I do not like collecting my PrEP pill at the clinic…umm*,* I do not go to the clinic”* *(RIYCRI003_P_C4-7M_F19).*

### Community based service providers

Our data suggest that challenges in accessing PrEP services at traditional health facilities influenced participants’ preference for decentralised service delivery. The PHC re-engineering model (Fig. 1) supports this approach by expanding PrEP access through local venues such as schools, pharmacies, community halls, and youth zones. It also promotes task-shifting, enabling pharmacists, lay providers, and Ward-Based Primary Healthcare Outreach Teams, such as community health workers and Linkage officials, to provide appropriate services. These decentralised providers were not only preferred but also actively used by PrEP users. The following section highlights how decentralising PrEP services, as recommended in the PHC re-engineering programme, benefited communities and explores the reasons behind these preferences.

#### Schools and colleges

Participants were comfortable with using colleges and secondary schools as service delivery points for PrEP. Many felt that schools would be ideal and acceptable to young adolescents, as they spend a significant amount of time there. They also believed that receiving services in this familiar environment could reduce the risk of discrimination, stigma, or judgment from peers.

##### Accessible (distance and transport cost)

Most of the senior students, especially boys, preferred schools because schools were accessible, they were everywhere, and a larger percentage of participants were in schools and colleges. One of the recognised benefits of schools as a PHC re-engineering service point is local presence,

*“The schools are close to all of us*,* we do not need to travel long distances*,* we do not need to pay money for transport.”* *(IMYCMS069_P_D4-7M_F_25).*

Participants believed that offering PrEP in schools would ensure easier access, as learners would not need to travel to health facilities. This aligns with the primary goal of the PHC re-engineering model, to improve access to essential healthcare services in under-resourced communities.

*“…it is better to access PrEP at the schools or pharmacy*,* or our college because these are there everywhere*,* most of the communities do not have clinics*,* you must travel long distance to the clinic*,* it is expensive.”* *(APTSUM044_P_C4_7M_M_24).*

##### Peer support

Psychosocial support was also valued by participants. Encouragement and support from other PrEP users, was reported as a facilitator to PrEP use as shown in quote below:

*“I also prefer the schools because I get support from my friends. There are many of us that are taking PrEP. We support each other. One of the teachers was also supporting us and encouraging us to take PrEP and protect ourselves.”* *(IMYCMS069_P_D4-7M_F_25).*

Schools play an important role in health promotion because adolescents spend most of their time in schools, which make them an ideal captive audience. Adolescents are not only easily accessible for preventive interventions in schools but can also support and encourage each other to take PrEP as shown in the quotes below:

*“It benefits us when PrEP is delivered in the community and schools. Youth can benefit as they can encourage each other. For example*,* if I see that my friend is taking PrEP I will follow her*,* but I cannot go to the clinic because youth do not use the clinic*,* they are young they do not get sick*,* so you will not see them at the clinic and will therefore not be there… So*,* I prefer schools*,* because I am already in school*,* and we all spend most of our time in schools and I also prefer in the community.”* *(MOCHMP119_P_I_F_22).*

*“I guess I can say at the school*,* because that is where the youth spend most of their time. yeah. When you play soccer*,* maybe also at school.”* *(IMYCMS042_P_I_M_21).*

##### Health promotion - Share information with friends

The schools also provide space/platforms for health promotion, sharing information, and peer education among adolescent learners as explained by this learner when she said:

*“…the good thing is that at the schools they tell us about PrEP in a group or in the assembly and other students are able to ask questions about PrEP and when they give us pills you can ask other question about the pills…”* *(RITSRI018_P_C4-7M_F_22).*

The young adolescents were reportedly comfortable receiving their PrEP pills among their peers who:

*“…did not judge or stigmatise anyone*,* everybody understands that we are boys …we are sexually active*,* and we need protection*,* even our teachers support us…”* *(IMYCMS140_P_D4-7M_M24).*

Seeing other peers in the school using PrEP to protect themselves also encouraged and motivated them to use PrEP.

*“It is better at the schools because no one will judge you*,* you see others also taking the PrEP pills to protect themselves*,* so you are comfortable to use since others are also using the pills.”* *(MACHMK088_P_D4-7M_F_22).*

The non-governmental organisations (NGOs) and the local clinic staff that worked on HIV prevention programmes also made use of the schools to do campaigns and promote PrEP uptake in rural communities. One adolescent explained that:

*“…they did not only use television … to tell people that there is something that can prevent and help people not to contract HIV…but the easiest way to do campaigns in the communities was to meet people in places like schools. In our school*,* they normally do it during prayer in the morning and talk about PrEP. The NGO guys and nurses ask for that short time*,* and they give us information about PrEP… I don’t know if they do that in other schools.”* *(IMYCMS062_P_I_M_18).*

*“…to support each other at schools and even in their communities*,* because a lot of times they spend more time with other young ones in their homes as compared to the adults. So*,* the youth can support and encourage each other to take PrEP and also convince others to initiate PrEP and they will be able to encourage those who don’t take PrEP for them to have the courage and enjoy the benefits of PrEP. advise them*,* advise them even when they are at home*,* or when in schools*,* have a proper discussion and explain more about PrEP.”* *(TAYCMS033_P_I_F_22).*

#### Pharmacies

Some participants supported the idea of using local pharmacies as PrEP collection points, noting that pharmacies are increasingly available in their communities. They highlighted several advantages, including accessibility, shorter or no queues, friendly and non-judgmental staff, and a sense of privacy and security. When asked about their preferred service provider, one adolescent responded:

*“They would prefer a pharmacy where they would go because for other people if you have a blue card*,* they automatically think you are HIV positive even if they did not ask what did you come for at the clinic. So that is why youth does not like to go at the clinic because if they are scared that they will say they have HIV while they do not have. I would prefer pharmacies because everyone goes there*,* if you go there*,* they will think you are buying something maybe medication*,* they will not know that you are buying or collecting PrEP pills…they don’t have queues*,* and it is here at the mall.”* *(IMYCMS062_P_I_M_18).*

*“It is also good at the pharmacy because it is not noticeable at the pharmacy. There is a lot of medication at the pharmacy…*,* and people will not know that you are collecting PrEP pills.”* *(TAYCMS033_P_I_F_22).*

The fear of judgment and stigma made pharmacies a more attractive and preferred option for young adolescents who wished to keep their PrEP use private:

*“Okay*,* the advantage of pharmacies is that everyone is allowed to go there*,* no one will know what you are there for. There is no blue card (referring to the HIV patients’ cards) …you are allowed to ask questions about the side effects of the pill and get assistance”* *(APTSUM124_P_I_M25).*

*“…the pharmacists do not ask a lot of questions*,* they just take the prescription and give you the medication you need*,* they will not judge you. It is the same when we buy condoms*,* they don’t ask you questions why*,* but the nurses ummh*,* they will put you in a courtroom (laughing)*,* they will ask you a lot of questions like a criminal”.* *(IMYCMS140_P_D4-7M_M24).*

The availability of PrEP pills at the pharmacies as compared to other PrEP service points was another key driver that influenced the young adolescents’ decision to prefer the pharmacy as a PrEP pills collection point.

*“In the pharmacies*,* they keep all types of medicine*,* so they can stock PrEP pills for us*,* and we can go and collect any time when our medication gets finished. They can give them the list of our names*,* and we can go and collect them. It will be easy because it is close to our home… at the clinic the pills get finished and my friend went there*,* and she was told they were out of stock.”* *(MOCHMP119_P_I_F_22).*

*“Well*,* pharmacies are also a good idea because we now have pharmacies in the shops close to us. At the pharmacy they do not ask questions*,* they just give you your pills.”* *(MACHMK088_P_D4-7M_F_22).*

#### Community halls

Community halls were also identified as preferred collection and service delivery points. Participants noted that quick and convenient access to PrEP services at these locations positively influenced their uptake. Adolescents emphasised that community-based HIV testing, and PrEP services were more accessible and efficient than nearby clinics. Many expressed that community halls offered a more comfortable and less intimidating environment for receiving care. One participant shared:

*“…the community halls*,* because it helps a lot of people who cannot afford transport*,* we can easily collect [PrEP pills] there…”* *(IMYCMS042_P_I_M_21).*

*“…the government must bring PrEP to the community and use community halls*,* most of the communities do not have clinics close to them*,* you must travel long distances.”* *(IMYCMS037_P_C4-7M_F_23).*

*“…think the boys will prefer…they are always at the grounds playing and we have a community hall next to the sports grounds. You can collect there when we finish our soccer match…”* *(CAYCMS020_P_I_F_23).*

Participants also suggested that community institutions like schools, churches and community halls should be used to distribute information and educate the community members about PrEP as there was limited knowledge on what PrEP is and how it works:

*“Yeah*,* I think like nurses have to go to schools uhm and other places like community halls and church and teach people over there*,* they should not wait for us at the clinics… youth do not go to the clinic…Yes*,* about how PrEP works because people don’t really know PrEP*,* like us we tested but we did not start PrEP*,* the clinic is very far.”* *(GOCHIM070_P_D4-7M_F_20).*

*“Most of the time you will find the youth playing soccer maybe in the street*,* so you ask them to come together and ask for their time and talk to them. Explain to them that there is such a thing called PrEP… maybe they don’t use protection when they have sex*,* so they will teach them about PrEP and what it does… So that anyone who is interested*,* you can take their contact details and contact them and give them PrEP. You cannot get the youth at the clinic*,* they don’t go.”* *(IMYCMS042_P_I_M_21).*

#### SECTION2Community health workers and support groups

Our data highlights the critical role that Community Health Workers (CHWs), and Linkage officials play in delivering primary health care services in the study community, as illustrated in the following quotes:

*“The community health workers help us and they also visit us regularly [to talk] about contraceptives. They also remind us when they come to see my baby for vaccination… We were visited by [the] PrEP people (stutter) after they tested us and gave us the PrEP [pills]*,* and they now just use the CHW who comes to check on us and provide the PrEP pills…”* *(APTSUM034_P_D4-7M_F_22).*

*“The young Linkage officials understand our problems. They collect pills [PrEP] for us and deliver to us; they teach us about PrEP and help us with side effects.”* *(IMYCMS037_P_C4-7M_F_23).*

*“We are comfortable with Community health workers because they always worked with the clinic*,* and they came here to give us condoms and contraceptives…I trust them. Now they are helping me not to skip taking my PrEP pills because they always come and deliver.”* *(PHHSMS036_P_C4-7M_M18).*

#### Support groups

Participants emphasised the need for support groups to foster mutual encouragement and long-term adherence to PrEP. These groups would provide adolescents with a platform to connect with fellow PrEP users, share experiences, and navigate challenges collectively:

*“The HIV guys have got some groups where they discuss about HIV. We need those groups for PrEP. We can support each other and share experiences about PrEP.”* *(IMYCMS021_2_P_I_F_21).*

*“It will help for people that are using PrEP to encourage each other. That they are encouraged by people that take PrEP*,* not people who do not take it and have never taken it. We need the PrEP groups and counsellors who have experience in PrEP.”* *(TAYCMS033_P_I_f_22).*

#### Youth zone

Participants also highlighted Youth Zones as effective locations for reaching young adolescents, noting that these spaces are commonly used for youth gatherings and discussions. They viewed Youth Zones as ideal platforms for sharing information about PrEP and as convenient, non-judgmental spaces for PrEP distribution.

*‘The youth zone will also be good because it is an area where youth meet to discuss issues that affect youth*,* they teach us and give us support. It is safe*,* no one judges you. They can bring our PrEP pills and teach us about PrEP.”* *(IMYCMS140_P_D4-7M_M24).*

*“I think it’s good for PrEP to be collected from the youth zone because most of the time no one else will know that you are collecting PrEP. I prefer youth zones. You will not be judged.”* *(TAYCMS033_P_I_F_22).*

#### Home delivery of medication

Home delivery of PrEP, facilitated by Linkage Officers, CHWs, and supporting NGOs/Non-Profit Organizations (NPOs), was seen as an effective way to improve access, especially for vulnerable adolescents. Participants cited convenience as a key benefit, noting it saved transport costs, reduced time spent traveling, and minimized nonadherence. This model aligns with the PHC re-engineering approach, offering a practical solution to common barriers in accessing facility-based PrEP services.

*“Delivering at homes*,* it is also good that they come closer because some of us are far from the hospital in the rural area. The clinics are far away (stutter) and the mobiles that usually come do not come often.”* *(MACHMK088_P_D4-7M_F_22).*

*“I think it’s a good idea to deliver PrEP pills at our home*,* it’s good because it may happen that you don’t have money to get to the clinic or the pharmacy*,* they can contact you and deliver it to you like it’s done for me…”* *(APTSUM003_P_C4_7M_F_19).*

*“We can benefit a lot*,* let say maybe a person is busy most of the time*,* and you do not get time to go and collect*,* they can deliver. You benefit because you do not miss taking your medication if it is delivered… and you do not have to go to collect it at the clinic sometimes*,* everything becomes easy. Every time I saw the CHWs*,* I got reminded to take my pill (laughing).”* *(CAYCMS020_P_I_F_23).*

*“Maybe a day could be arranged where cars would come and deliver PrEP to us*,* in our homes… Especially men*,* they fear being seen collecting PrEP at the clinic…”* *(MOCHMP199_P_D4-7M_F_19).*

### Preference of integrated services

Several participants also expressed a preference for integrated services, emphasising the convenience. Their socio-economic living conditions and challenges in accessing health services often made it difficult for some of them to make repeated trips to the same health facility for different services. The majority of our participants were still in school or college and faced competing demands related to their studies and daily responsibilities, which limited the time and resources available for multiple healthcare visits. Consequently, participants indicated a strong preference for being able to collect their PrEP pills alongside other medication. The excerpts below illustrate participants’ reasons for favouring integrated health service delivery:

*“You know we come from poor families and our parents struggle to pay fees and my upkeep money at college…you know I went for my appointment to collect my PrEP pills and I asked the nurse to give me contraceptives*,* she said I should go and queue in the other room with the nurse for that…the queue was very long in that room and I had to go home because I was late for my classes. I had to ask for money to go back again*,* it is not good”* *(TAYCMS033_P_I_F_22).*

I prefer that they just give us everything we need when we go to the clinic instead of going back many times, it is very expensive to travel many times you see. I don’t have that time, I am busy at school…*(MOCHMP017_P_D4-7M_F_22)*.

*“Because I only get PrEP here at Maritzburg*,* I do not even know how to access it that side (Stanger). So perhaps they could do this thing that maybe you are a ‘clinic person’*,* like you are a person who collects chronic medication at the clinic*,* I live in Maritzburg*,* and I relocate to Stanger*,* then I can use the same card. Then I would be able to use the same card to collect my chronic medication on the other side* *(IMYCMS069_P_D4-7M_F_25)”.*

Furthermore, some young adolescents expressed the desire for a more flexible, hybrid system that would allow them to choose the methods of receiving the pills that best suits their needs. They emphasised that the decision should be theirs to make, with respect for their preferences to encourage greater participation in the PrEP programme. As one participant explained:

*“However*,* if it is delivered to you*,* it can also be fine. The decision lies within a person and [should] be supported… If the decision is respected*,* they can be able to talk to others and say hhaybo [No way!]*,* you can maybe ask how it is delivered … I wish they can allow us to choose how we can get the pills*,* you say it yourself what you want… and where you want to collect [the pills] …and they respect your decision; they do not do things without your will; I think that that will encourage people.”* *(RIYCRI003_P_C4-7M_F19).*

## Discussion

This study examined young people’s perceptions and experiences regarding the decentralisation of PrEP services through the Primary Health Care (PHC) re-engineering structures and community-based approaches to improve the initiation, continuation, and adherence to oral PrEP. The findings highlight several critical factors that contribute to the successful implementation and expansion of PrEP services among high-risk, sexually active adolescents and young adults (AYAs) in South Africa. In alignment with the PHC re-engineering programme [12] the study underscores the need for decentralised PrEP services to enhance uptake, adherence, and overall effectiveness.

Decentralisation emerged as a key strategy for increasing both PrEP uptake and adherence, particularly among AYAs. The study found that decentralised services, such as the use of community-based facilities, outreach teams, and home delivery systems, were particularly appealing to the young adults because they addressed major barriers, including cost, distance, and stigma [28]. Some participants highlighted how home delivery of PrEP not only saved time and money but also supported adherence to the medication. These findings underscore the potential of decentralised PrEP delivery to significantly improve adherence and treatment outcomes for at-risk youth [29, 30]. Similar findings were reported by Mataboge and others (2023), in Gauteng Province, South Africa, where participants expressed a clear preference for decentralized and simplified service delivery models that facilitated access to health services [31]. Likewise, other studies have demonstrated that young people preferred decentralised PrEP service delivery points such as pharmacies, youth zones as these models improve both PrEP access and uptake [32, 33].

Participants emphasised the accessibility of services at community health clinics and youth zones, which provided a more convenient and private environment for accessing PrEP. Many young people reported feeling more comfortable collecting PrEP from community-based points compared to traditional healthcare facilities, which are often associated with long waiting times, overcrowding, and fear of judgment [30]. Additionally, community-based services helped mitigate transportation costs and the logistical challenges of travelling to distant healthcare facilities, an especially important consideration for AYAs living in rural or underserved areas.

### Comparison of different PrEP service delivery points (SDPs)

This study found varying preferences for PrEP SDPs. While traditional clinics remain a standard SDP, many participants preferred more discreet, youth-friendly spaces like community youth zones, schools, and pharmacies. Youth zones were especially preferred for providing a safe, judgment-free environment to access services. The integration of PrEP services into these zones allowed young people to collect their medication in an environment where they felt comfortable and free from judgment. Integrating PrEP into such spaces improved comfort and accessibility [34, 35]. School-based health programmes were also seen as effective, leveraging the time adolescents spend in schools to deliver scalable, cost-effective HIV prevention [36, 37].

Pharmacies offered a discrete, less stigmatized option compared to clinics, making them a promising alternative [38]. These findings highlight the need for multiple, flexible access points tailored to young people’s preferences and lifestyles [39]. Decentralised, user-centred models, such as those offered in schools, youth zones, and pharmacies, can significantly improve PrEP uptake and sustained adherence by meeting adolescents where they are, both physically and socially [29, 40].

### Encourage community participation and support

The involvement of the community, particularly through community health workers (CHWs) and peer support groups, is a key element of the PHC re-engineering programme [12]. Our data highlights the significant role CHWs play as familiar and trusted figures within the community. They extend health services beyond the individual patient as they provide services to people in their homes as prescribed in the Government of South Africa, 2018 policy framework for WBPHCOTs [41]. They facilitate access to PrEP services by linking young people, providing medication reminders, and offering essential information about PrEP. By utilizing this important cadre of healthcare workers, the programme strengthens the broader PHC system at the community level.

This approach creates a positive cycle, enhancing healthcare services for underserved households and ensuring easy access to PrEP. This peer-like support system not only facilitated access to PrEP but also improved medication adherence by tracing defaulters and link them back to treatment, offering emotional support and guidance on managing side effects [18]. The long-term persistent problem of stigma and confidentiality was however raised as a concern by some adolescents and young adults in this study and other studies who feared that CHWs might share information about them to neighbours or other family members [19].

While decentralisation holds immense promise for expanding access to PrEP services, it also presents a range of challenges that may affect both the sustainability and effectiveness of services delivery. Although decentralisation has been proven to bring health services closer to communities [33], it can paradoxically heighten concerns about confidentiality and stigma. In the current study, which was conducted in small close-knit communities where HIV remains highly stigmatised, some participants expressed fears of being recognised or judged for using PrEP by schoolmates, neighbours, or community members. A perceive lack of privacy was further undermined the acceptability of community-based PrEP services.

Evidence from other studies support these findings, indicating that some of the decentralised PrEP services lack strong linkages to care, and/or structured follow up, which may contribute to PrEP discontinuation due to side effects, forgetfulness, or changes in perceived risk [42, 43]. To address these challenges, decentralised services should be implemented with other innovative adherence support tools such as SMS and WhatsApp reminders, Facebook peer support groups, monthly phone calls, and weekly messages to enhance continuation of PrEP use and user engagement [32].

Furthermore, peer support groups, which have historically been instrumental in supporting individuals living with HIV, were highlighted as a vital resource for promoting adherence and engagement among PrEP users. Our findings suggest that sharing experiences within these groups could foster a sense of solidarity, reduce stigma, and improve adherence by empowering individuals with the support they need to navigate their PrEP journey [44].

### Opportunities for peer engagement and support

Findings from our study highlight the critical role of peer engagement in promoting PrEP uptake and adherence among young people. Participants noted that adolescents and young people are more likely to seek information about PrEP from peers who have firsthand experience rather than from a healthcare professional or family member, who may not be as knowledgeable or supportive [45, 46]. Peer-led support groups provide a platform for open discussions about challenges and successes [47], helping to normalise PrEP use among young people, while reducing the stigma often associated with HIV prevention.

Furthermore, peer counsellors and educators play an essential role in improving PrEP adherence [49]. As trained individuals with lived experiences, they are viewed as relatable and trustworthy sources of information. Their ability to provide tailored guidance, dispel misconceptions, and offer ongoing support can significantly enhance the effectiveness of PrEP services. By fostering a sense of community and shared responsibility, peer-driven initiatives can strengthen engagement with PrEP and contribute to better health outcomes for AYAs [48].

### Integration of services at community level

Integrating PrEP into broader community-level sexual and reproduction health (SRH) services was a key theme in our study. Adolescents emphasized the need for comprehensive, people-centred services combining HIV prevention, sexual education, contraception, and peer support. Such integration promotes a holistic, coordinated approach to adolescent health [49], enhancing engagement with PrEP and other HIV prevention tools across different care levels and beyond health facilities [37, 50].

Our findings suggest that integrating condoms and contraceptives with PrEP services promotes safer sex practices and reduces the risks of HIV and unintended pregnancy. This aligns with community-based models like the Tutu Teen Truck in South Africa, which offer multiple delivery options, to address access and privacy barriers to PrEP uptake [35]. Personalised service delivery is crucial for the success of PrEP initiatives, as traditional clinical settings often discourage users due to stigma and logistical challenges. Integrating SRH and HIV prevention service at the community level helps address broader social health determinants, such as access to information, education, and affordable healthcare [51].

Partnerships with NPOs at community level support healthcare teams through mobilisation, service delivery, and health education. Similar studies highlight the role of partnership in facilitating community entry and addressing local challenges [52]. Additionally, users also report anxiety when refilling PrEP in unfamiliar settings, reinforcing the need for hybrid models combining community- and facility-based services to enhance engagement and continuity of care for better engagement [53].

### Potential for increased engagement with and uptake of HIV prevention and SRH services

Decentralising health services through the PHC re-engineering model plays a critical role in reaching underserved populations in remote areas, were physical distance and socio-economic barriers limit access to healthcare facilities [13]. In our study, adolescents emphasized that quick, convenient access to community-based HIV testing and PrEP services significantly influenced their uptake. Services offered through schools, youth zones, pharmacies, and even home delivery better aligned with their daily schedules and needs, making them preferable to traditional clinic-based models.

However, challenges with facility-based PrEP services persist, despite some youth expressing a preference for this delivery model. Participants cited long travel distances, transport costs, stigma, negative provider attitudes, low PrEP awareness, and a lack of youth-friendly spaces as major deterrents. Some indicated that family members confused PrEP with lifelong ART, while others reported discomfort discussing sex-related issues due to cultural and religious norms [16] that shape provider biases, often resulting in judgment or denial of care. Inadequate clinic preparedness, poor infrastructure (e.g. use of park homes), and staff attitude towards young adolescents were also listed as challenges in accessing PrEP services.

Adolescents and young adults (AYAs), in particular, face greater barriers to PrEP uptake and adherence, contributing to continued high HIV-related mortality in this group. Traditional service delivery models, often designed by adults, fail to reflect the realities of youth, making them less effective [11, 34]. There is an urgent need for youth-centred innovations that improve service delivery, retention, and health outcomes. Meaningful youth engagement in designing and implementing interventions is essential to ensure services are relevant, accessible, and effective. Evidence from several low-income and middle-income countries (LMICs) supports the potential of such approaches, although outcomes have varied, underscoring the importance of context-specific solutions [54].

### Policy implications

This study offers critical insights into the provision of decentralised PrEP, providing a valuable contribution to both PHC re-engineering model and public health interventions in resource constrained environments. It underscores the importance of developing policies that enhance HIV prevention efforts for AYAs in South Africa, with a specific focus on integrating PrEP into PHC services. This is particularly significant given the challenges faced by adolescents and young adults in high HIV prevalent communities, where access to sexual and reproductive health services are often limited. The findings underscore the need for a comprehensive PHC re-engineering model that accommodate the challenges of AYAs to ensure the programme’s sustainability, offering actionable recommendations that can inform the broader design and delivery of similar initiatives at community level. Policies must prioritise youth-friendly, community-based approaches to ensure accessibility, sustainability, and equity.

Community health workers (CHWs) are essential in linking health systems with communities, particularly for young people who face barriers such as stigma, cost, and distance. This has demonstrated that the PHC re-engineering programme has the potential to enhance both coverage and quality of health services [12]. CHWs’ local knowledge, trust within communities, and ability to deliver services beyond health facilities position them as key agents in HIV prevention. Policies should strengthen CHW programmes through formal recognition, adequate training, fair compensation, and support. Their role in promoting adherence and tracing defaulters should be embedded in retention strategies for youth on PrEP.

This study highlights the need for integration of PrEP into broader sexual and reproductive health (SRH) services, including family planning and sexual health education in order to provide a holistic approach to HIV prevention. Expanding delivery through youth-friendly clinics, schools, mobile units, pharmacies, and community centres will help address access barriers such as transportation and privacy concerns.

Finally, engaging youth in the design and implementation of HIV prevention programmes and incorporating ongoing feedback mechanisms will help ensure that services remain responsive, relevant, and effective.

### Limitations

Several limitations should therefore be considered when interpreting the results and applying them to other contexts. First, the study only focused exclusively on adolescents and young adults identified as being at high risk of HIV infection thereby excluding AYAs who were not classified as high risk. Secondly, the study was conducted in a single district in KwaZulu Natal, and the relatively small, geographically concentrated sample may not reflect the broader perspectives and experiences of AYAs accessing decentralised PrEP service delivery points in other districts or provinces.

Additionally, while the qualitative methods provided detailed contextual insights into participants’ experiences and perspectives, it does not allow for statistical generalisation or capture the full diversity of views across all AYAs subgroups. Our findings may not be generalizable to other populations that are using PrEP and are using different service delivery points. The findings should therefore be interpreted within the specific social and cultural context in which this study was conducted.

Future research could benefit from a larger and more diverse sample across multiple settings, as well as the integration of various data collection methods to strengthen the reliability, validity and generalisability of the results.

## Conclusion

This study highlights the critical role of decentralised, community-based approaches in improving PrEP uptake, adherence, and continuation among AYAs in South Africa. The findings underscore the importance of integrating PrEP services into broader SRH services, addressing key barriers such as distance, stigma, and accessibility, and utilizing CHWs and peer support systems to enhance engagement and retention. These strategies are particularly vital for high-risk groups, including AGYW and ABYM, who face unique challenges in accessing and adhering to PrEP.

By decentralising PrEP services and integrating them within familiar, youth-friendly spaces, such as schools, youth zones, community halls, and mobile clinics, this study demonstrates how HIV prevention can be made more accessible and acceptable to young people. Additionally, the involvement of peers and CHWs as key facilitators in PrEP service delivery provides a trusted support system, reducing stigma and fostering a sense of solidarity among users.

There is a need for comprehensive, youth-centred interventions, and the expansion of community-based models to ensure that PrEP reaches those who need it most. It further demonstrates the importance of supporting and scaling up PHC re-engineering model, a proactive approach that takes healthcare to the people, in their homes and communities. To effectively address the HIV epidemic among AYAs, policymakers must prioritize the development of inclusive, adaptable, and accessible service delivery models that respond to the specific needs of this demographic.

In conclusion, the findings call for continued innovation and investment in decentralised, integrated, and youth-centred PrEP delivery systems to reduce HIV transmission and improve health outcomes for South Africa’s most vulnerable populations.

## Supplementary Information

Supplementary Material 1.

Supplementary Material 2.

## Data Availability

The datasets used and/or analysed during the current study are available from the corresponding author on reasonable request.

## Abbreviations

ABYM: Adolescent boys and young men
AGYW: Adolescent girls and young women
ART: Antiretroviral treatment
AYAs: Adolescents and young adults
CHWs: Community health workers
DREAMS: Determined, Resilient, Empowered, AIDS-free, Mentored, and Safe
HCPs: Healthcare providers
HIV: Human Immunodeficiency Virus
IDIs: In-depth interviews
KZN: KwaZulu-Natal
LMICs: Low- and middle-income countries
NHI: National Health Insurance
NRF: National Research Foundation
NGOs: Non-Governmental Organizations
NPOs: Non-Profit Organizations
PEPFAR: U.S. President’s Emergency Plan for AIDS Relief
PHC: Primary health care
PrEP: Pre-exposure prophylaxis
SAMRC: South African Medical Research Council
SDPs: Service delivery points
SRH: Sexual and reproductive health
SDPs: Service delivery points
US: United States
WBPHCOTs: Ward-based Primary Health Care Outreach Teams
