# Supplementary material for: A primary health care Re-Engineering approach to enhance PrEP initiation and adherence among high-risk, sexually active adolescents and young adults in KwaZulu-Natal, South Africa
Source: BMC Public Health. 2025 Dec 22;25:4308. doi: 10.1186/s12889-025-25698-2 (PMC12751761; doi:10.1186/s12889-025-25698-2)
Supplement: Supplementary file 1 — Supplementary Material 1. [file 12889_2025_25698_MOESM1_ESM.docx]

**Supplement A: In-depth interview guide**

| **Category** | **Interview questions** |
| --- | --- |
| **Awareness** | **Questions for participants who initiated/ continued/discontinued PrEP**  **Identifying individuals at highest risk for contracting HIV**  Do you feel you can control or reduce the risks of contracting HIV? (Elaborate)  What are some of the things that you think if you do, could put you at risk of contracting HIV? (Elaborate)  What do you do to reduce the risks of contracting HIV? |
|  | **Questions for participants who did not initiate**  **Increasing HIV risk awareness among those individuals**  Have you ever heard of PrEP? *(Probe: where did you first hear of it)*  What is your understanding of PrEP? (Elaborate)  Do you know anyone who uses PrEP?  How do you think PrEP could help to reduce HIV risks? |
|  | **Questions for participants who initiated/did not initiate/continued/discontinue d**  **Enhancing PrEP awareness**  What are some of the ways that you think could be effective in increasing knowledge about PrEP?  What are the other ways you think the information on PrEP could be shared? |
| **Access** | **Questions for participants who initiated/continued**  **Facilitating PrEP access**  What kinds of messages should be included in counseling clients to decide if they want PrEP?  Which people would benefit from PrEP?  Where do you think young people like you would prefer to go for PrEP services and to collect PrEP pills? (Probe for health clinic, ARV clinic, youth-friendly clinic/service, pharmacy, private provider, family planning clinic, NGO) What are the pros and cons of each place?  What do you think about the idea of PrEP being delivered outside of a health facility (such as at school or at your home)?  What might be the barriers and benefits to this different approach?  What are the factors that can make it difficult for a person to access PrEP and why?  To what extent would people in your community support you to take PrEP? *(Probe: Why or why not?)*  cf |
|  | **Questions for participants who initiated PrEP**  **Linking to PrEP care**  I understand that a health worker recently recommended that you to start taking pre-exposure prophylaxis (PrEP) pills to help prevent HIV. Can you tell me what the conversation you had with the health worker was like?  What kinds of information did the health worker share with you about PrEP?  Did you have any questions that the health worker couldn’t answer?  /How comfortable did you feel speaking with the health worker about PrEP?  What was your understanding of PrEP before this appointment?  What type of health providers are best to provide PrEP services to young people like you? (*Probe: Why and whether age and gender matter)*  Are you part of any community interventions/clubs/groups? (*Probe: What are the names of the groups/interventions you participate in? What are the main activities happening in your club/group? How did these interventions help you to initiate/not initiate PrEP?)* |
|  | **Questions for participants who initiated PrEP**  **Prescribing PrEP**  What worries or concerns did you have about starting PrEP?  If you were going to create a campaign to encourage young women or men like yourself to consider using PrEP to reduce their risk of getting HIV, how would you describe what PrEP is and how it works (in your own words)?  What information about PrEP should be emphasized in communication campaigns to young women and men? What should not be emphasized?  What do you think potential PrEP users would need to stay on PrEP (in the form of reminders, support, etc.)? |
| **Uptake** | **Questions for participants who initiated/continued on PrEP**  **Initiating PrEP**  What were the main factor that made you decide to start taking PrEP?  Why do you think people might be interested in starting PrEP?  What are some of the emotional benefits of PrEP that you can think of?  What do you think would help adolescents and young women/adolescent boys and young men to take and stay on PrEP?  What do you think can be done to make PrEP more acceptable as an HIV prevention method?  What were some of the other issues you considered when you decided to take PrEP?  Is there anything that might make you change your mind about PrEP? |
| **Adherence** | **Questions for participants who initiated/continued on PrEP**  **Adherence to doses prescribed or minimum effective doses**  What are some of the issues you have been facing with taking your PrEP? (*Probes: support from family, peers, partners, heath providers)*  What motivates or demotivates you regarding taking your PrEP?  To what extent have you been able to integrate PrEP into your life? What do you think are some of the things that could make you stop taking your PrEP?  Are there any ways that you think health workers can help you to overcome the challenges you talked about? |
| **Continuation** | **Questions for participants who Initiated/continued on PrEP**  **Retaining individuals in PrEP care**  How long do you think you would like to take PrEP? *(Probe: Why do you think it is important for you to take it that long?)*  What is motivating you to take your medication every day and for that long?  In what ways can the use of PrEP affect your sexual relationships and your relationships with family members and friends?  If your friends, sexual partners and members of your family (parents) were to support your use of PrEP, what form will you desire this support to take?  If you were to discontinue PrEP, what would be the possible reasons |
| **Discontinuation** | **Questions for participants who did not initiate/discontinued on PrEP**   - **PrEP uptake decisions**: What was the main factor that made you decide not to start taking PrEP? What were some of the other issues you considered when you decided not to take PrEP? Is there anything that might make you change your mind about PrEP? - **Programmatic suggestions**: The government is going to be offering PrEP for free in public health clinics. Do you have any suggestions for how the delivery of PrEP can be improved? What kinds of messages should they include in counselling clients to decide if they want PrEP? Are there any ways that you think health workers can help you to overcome the challenges you talked about today? |
