# Supplementary material for: A primary health care Re-Engineering approach to enhance PrEP initiation and adherence among high-risk, sexually active adolescents and young adults in KwaZulu-Natal, South Africa
Source: BMC Public Health. 2025 Dec 22;25:4308. doi: 10.1186/s12889-025-25698-2 (PMC12751761; doi:10.1186/s12889-025-25698-2)
Supplement: Supplementary file 2 — Supplementary Material 2. [file 12889_2025_25698_MOESM2_ESM.pdf]

13 December 2021

Dr Edward Nicol
Burden of Disease Research Unit
SAMRC Cape Town

Dear Dr Nicol

**Protocol ID:**	EC051-11/2020
**Protocol title:**	Strengthening Health System's Capacity for Pre-Exposure Prophylaxis for AGYW and ABYM in South Africa (SHeS'Cap PrEP Study)
**Meeting date:**	29 November 2021

Thank you for your submission to the Committee, dated 19 November 2021, to re-apply for approval to include participants aged 15 to 17 years with a waiver of parental consent in line with the SOP 'Conduct of Research involving Minors – Waiver of Parental Consent' released on 4 November 2021.

The Committee stressed that the biggest concern and the biggest risk regarding waiver of parental consent is when situations should arise where mandatory reporting is necessary, in which case the research will come to the attention of the parents/legal guardians. The parent/legal guardian could then take it to the Children's Court. Such cases must be brought to the Committee's attention immediately for the Committee to assist in mitigating the risk.

The Committee agreed that now that the SOP has been developed, this request for waiver of parental consent is approved for 15-17-year-olds with the following provisos:

1. Should a case of mandatory reporting arise in terms of the relevant legislation (the Children's Act and the Sexual Offences Act), it must be reported to the Committee within two business days as per the Committee's SOPs, i.e. the incident of mandatory reporting will be treated much like an SAE. The Committee has the right to then suspend the research if deemed necessary. A social worker or the Dept of Social Development must be involved.
2. Six-monthly reports must be submitted with special attention to:
   - The number of participants recruited under the waiver,
   - The reason the waiver was applied in each case, and
   - Incidents of mandatory reporting (which would have already been sent to the Committee in terms of the point above).
3. The researchers should inform the Committee when the research will commence and indicate accordingly the dates when they will be submitting the 6-monthly reports.

THE SOUTH AFRICAN MEDICAL RESEARCH COUNCIL
Francie Van Zijl Drive, Parowvallei, 7505, Cape Town | Po Box 19070, Tygerberg, 7505, South Africa
Tel: +27 (0)21 938 0687 | Fax: +27 (0)86 685 4023 | Email: adri.labuschagne@mrc.ac.za | Web: www.samrc.ac.za/ethics/ethics.htm

Yours sincerely

Prof Danie du Toit
Prof Danie du Toit
Chairperson: SAMRC Human Research Ethics Committee

Members present at the meeting: Prof D du Toit (Chairperson), Ms S Behardien, Adv J Early, Dr H Etheredge, Ms M Ledwaba, Prof C Lombard, Dr W Zembe

THE SOUTH AFRICAN MEDICAL RESEARCH COUNCIL
Francie Van Zijl Drive, Parowvallei, 7505, Cape Town | Po Box 19070, Tygerberg, 7505, South Africa
Tel: +27 (0)21 938 0687 | Fax: +27 (0)86 685 4023 | Email: adri.labuschagne@mrc.ac.za | Web: www.samrc.ac.za/ethics/ethics.htm
